# Long Non-coding RNAs: A New Regulatory Code for Osteoporosis

**DOI:** 10.3389/fendo.2018.00587

**Published:** 2018-10-04

**Authors:** Qian-Yuan Wu, Xia Li, Zong-Ning Miao, Jun-Xing Ye, Bei Wang, Feng Zhang, Rui-Sheng Xu, Dong-Lin Jiang, Ming-Dong Zhao, Feng Lai Yuan

**Affiliations:** ^1^Third Affiliated Hospital of Nantong University, Nantong, China; ^2^Jinshan Hospital, Fudan University, Shanghai, China

**Keywords:** long non-coding RNAs, bone remolding, osteoblasts, osteoclasts, osteoporosis

## Abstract

Osteoporosis is a metabolic bone disease characterized by a decrease in bone mass and degradation of the bone microstructure, which increases bone fragility and fracture risk. However, the molecular mechanisms of osteoporosis remain unclear. Long non-coding RNAs (lncRNAs) have become important epigenetic regulators controlling the expression of genes and affecting multiple biological processes. Accumulating evidence of the involvement of lncRNAs in bone remolding has increased understanding of the molecular mechanisms underlying osteoporosis. This review aims to summarize recent progress in the elucidation of the role of lncRNAs in bone remodeling, and how it contributes to osteoblast and osteoclast function. This knowledge will facilitate the understanding of lncRNA roles in bone biology and shed new light on the modulation and potential treatment of osteoporosis.

## Introduction

Osteoporosis is the most common type of skeletal disease with weakened bone, clinically characterized by low bone mass and associated with increased fracture risk (1). The rising number of fractures due to osteoporosis and their associated morbidity place heavy burdens on public health services (2). As a larger proportion of the population reaches advanced age, the annual incidence rate and cost of osteoporosis-related fracture in the United States are predicted to increase by 48% by 2025, reaching almost 3 million fractures and ~ $25.3 billion in healthcare spending (3). This prognosis has prompted global efforts to find more effective means of osteoporosis treatment and prevention.

In people with osteoporosis, bone loss is due to increased osteoclast activity, and decreased osteoblastic bone formation cannot keep pace with bone resorption (4, 5). Most existing therapeutics for osteoporosis are antiresorptive drugs, such as bisphosphonates, and anabolic medications, including denosumab and teriparatide (6). Although these drugs effectively inhibit osteoclast activity, they also, suppress bone remodeling, which decreases bone quality and increases skeletal fragility and the risk of bisphosphonate-related osteonecrosis of the jaws (7). Given these many adverse factors related to current treatments, the exploration of novel anabolic therapeutic targets, and biomarkers for early detection and treatment is a primary goal in the field.

Recently, long non-coding RNAs (lncRNAs) have been found to play important roles in regulating various cellular processes, including those of bone cells (8, 9). LncRNAs form a novel class of non-coding RNAs; their chain lengths exceed 200 bases and they cannot be translated into proteins (10). Recent research has revealed that lncRNAs can shuttle to various subcellular locations and induce alterations across various stages of cellular development and differentiation (11). Although several lncRNAs have been proven to play significant roles in the regulation of bone remodeling, most lncRNA functions in bone cell biology are poorly characterized. Thus, we conducted this review of the current literature on the roles of lncRNAs in bone remodeling, with a focus on novel findings regarding lncRNA regulation of osteoblasts and osteoclasts. The review provides new insight into the modulation and treatment of osteoporosis.

## Sources and functions of lncrnas

### LncRNAs: an overview

LncRNAs have transcript lengths ranging from 200 nt to 100 kb and exist widely in nuclei and cytoplasm (12). As they lack open reading frames, they almost never participate in protein coding; instead, they regulate gene expression at the RNA level (13). Compared with research on protein coding sequences and small RNAs, lncRNA research is still in development, but it has already revealed a wide range of molecular biological functions for this non-coding RNA class. Approximately 15,779 lncRNAs in the human genome have been recorded (https://www.gencodegenes.org/, 05/2018).

LncRNA is similar to messenger RNA (mRNA) in many aspects. For example, it is usually produced by RNA polymerase II; the transcript has a 5′-cap and a polyadenylation structure. Therefore, lncRNA has been called mRNA-like long-chain RNA. Computer analysis has shown that lncRNAs have small numbers of start codons and reading frames, and that they are similar in structure and sequence to the 3′-untranslated regions encoding protein RNAs. LncRNAs have obvious tissue and space–time specificities (14). However, compared with protein coding genes, lncRNAs not only have little or no protein-coding function, but also low transcription levels, low sequence conservativeness among species, and little evolutionary pressure (15).

Compared with microRNA (miRNA), lncRNAs have longer sequences, more complex spatial structures, and more action modes (16). MiRNA functions can be performed by complete or incomplete base pairing with target genes; thus, miRNA targets are easier to find than lncRNA targets. Moreover, miRNA has been studied for a long time, producing relatively standard nomenclature and more complete databases, which is more conducive to bioinformatics and functional analyses. In addition, although quantitative polymerase chain reaction detection of lncRNA does not require stem-loop primers to elongate the sequence as for miRNA, lncRNA degrades more easily due to its low structural stability compared with mRNA. Thus, the extraction of lncRNA from RNA is more challenging (17).

### LncRNA categorization

According to the distance between lncRNA and the protein coding sequence, as well as their relative positions, lncRNAs can be divided into five categories: (1) synonymous lncRNA, in which one or more exons overlap with another transcript in the same strand; (2) antisense lncRNA, in which one or more exons overlap with another transcript in the complementary (antisense) strand; (3) bidirectional lncRNA, in which the starting point for expression is very close to those of adjacent coding transcripts in complementary strands; (4) intron lncRNA, in which an intron is derived entirely from another transcript; and (5) inter-gene lncRNA (or lincRNA), located in the interval between two genes. In addition, lncRNA can be divided into four categories according to function: signaling molecules, decoy molecules, primers, and framework molecules (18, 19).

### Common functions and mechanisms of action

Many studies have shown that lncRNAs play important roles in many biological processes (20). The main functions of lncRNAs are: (1) to impact downstream gene expression by inhibiting RNA polymerase II or mediating chromatin remodeling and histone modification; (2) to interfere with downstream gene expression by transcription in the upstream promoter region of protein-coding genes; (3) to generate various cleavage forms by forming complementary double strands with mRNA, and to interfere with mRNA splicing; (4) to regulate gene expression levels by forming complementary double strands with transcripts of the protein-coding gene and endogenous small interfering RNA (siRNA) under the action of the Dicer enzyme; (5) to regulate the activity of corresponding proteins; (6) to perform transcription as precursor molecules to small RNA molecules, such as miRNAs and piwi-interacting RNAs; (7) to change the cytoplasmic localizations of specific proteins by binding to them (21).

### LncRNAs in bone remodeling

Bone mineral density increases rapidly during puberty, and peak bone mass is achieved around 10 years after the completion of bone growth (22, 23). In adults, bone mass is maintained at a homeostatic level by continuous replacement of old bone tissue with new tissue, which is the foundation of dynamic bone remodeling. An increase in bone resorption caused by impaired bone formation results in bone loss. Bone loss is natural with aging, but it can also be exacerbated at a relatively young age by increased osteoclast activity, leading to bone injury (24). Increased osteoclast activity is seen in postmenopausal osteoporosis (PMOP), autoimmune diseases (25), Paget's disease (26), cancer (27), and type 1 diabetes (28, 29). Bone loss is also a side effect of the use of certain drugs, including glucocorticoids, thiazolidinediones, and H2 receptor antagonists (30), and it is considered to be an important secondary risk factor for fracture and secondary osteoporosis.

Osteoblasts, osteoclasts, and osteocytes, the main cells involved in bone remodeling, are regulated tightly by various systemic and local factors, such as cytokines, hormones, growth factors, the immune system, mechanical loading, and extracellular acidosis (31–33). During the formation of bone tissue, the Wnt/β-catenin and transforming growth factor (TGF)-β/ bone morphogenic protein (BMP) pathways modulate the Runt-related transcription factor 2 (RUNX2) to induce the osteogenic phenotype (34). The transcription factor osterix is required for osteoblast differentiation (35). The macrophage colony-stimulating factor and receptor activator of nuclear factor kappa B ligand send key signals inducing osteoclast development (35).

### Key lncRNAs in osteoblasts

**Figure 1:**
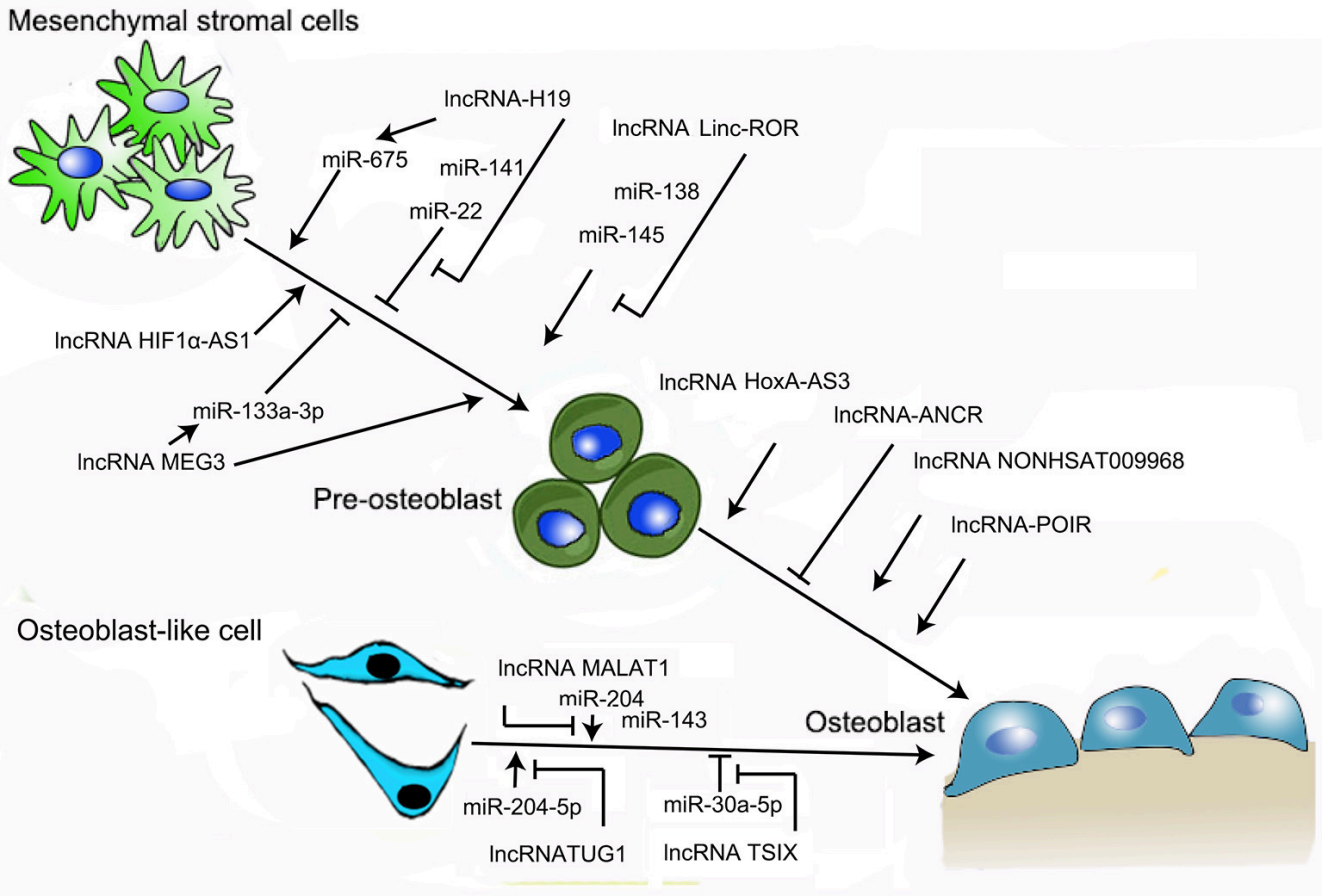
Schematic of the role of lncRNAs in the regulation of osteoblast proliferation. LncRNA-H19, lncRNA–HIF1α-AS1, lncRNA TUG1, lncRNA MEG3, lncRNA MALAT1, lncRNA Linc-ROR, lncRNA HOXA-AS3, lncRNA NONHSAT009968, and lncRNA-POIR are likely involved in this process. In addition, lncRNA-H19 indirectly induces osteoblast proliferation via targeting of miR-675. Potent inhibitors of osteoblast proliferation include lncRNA-ANCR, lncRNA-H19, lncRNA MEG3, and lncRNA TSIX. Furthermore, lncRNA-H19, lncRNA MEG3, and lncRNA TSIX attenuate osteoblast proliferation indirectly via miRNA.

Osteoblasts not only play a critical role in bone formation by synthesizing multiple bone matrix proteins, but also induce osteoclast development, resulting in bone resorption, through soluble factors and cognate interaction (36). A series of studies has demonstrated that various lncRNAs are implicated in the regulation of osteoblast proliferation and function via gene targeting at the transcriptional, post-transcriptional, and epigenetic levels, as well as via competing endogenous RNA (ceRNA; Figure 1) (37–41).

### LncRNA anti-differentiation non-coding RNA

Anti-differentiation non-coding RNA (ANCR) is a novel lncRNA that is down-regulated during stem cell differentiation, which is necessary to keep osteoblasts in an undifferentiated state. The first identification of lncRNA-ANCR was in association with osteoblast differentiation (37). Recently, siRNA-targeting lncRNA-ANCR was found to increase the levels of osteoblast differentiation markers, such as alkaline phosphatase (ALP) and osteocalcin (OCN), whereas overexpressed lncRNA-ANCR was found to induce decreased expression of these markers in osteoblasts. Mechanistically, lncRNA-ANCR has been shown to regulate the expression of RUNX2 by recruiting zeste homolog 2 (EZH2), which catalyzes H3 lysine-27 trimethylation in RUNX2 gene promoters, causing the inhibition of Runx2 and subsequent osteoblast differentiation.

RNA pulldowns have shown a direct association of ANCR with EZH2, but no evidence indicates that ANCR binds directly to or recruits EZH2 to bind directly to the RUNX2 promoter. This topic is of interest for future investigation. Recent studies have also demonstrated that the down-regulated level of lncRNA-ANCR promotes osteoblast differentiation of periodontal ligament stem cells (PDLSCs) by up-regulating osteogenic markers such as ALP, sialophosphoprotein, OCN, bone sialoprotein, and RUNX2 (42). LncRNA-ANCR is believed to control osteogenic differentiation of PDLSCs associated with the canonical Wnt signaling pathway. However, deeper evidence for the interrelationship between lncRNA-ANCR and this pathway is lacking; future studies should investigate this interaction.

During osteogenic differentiation, expression of the lncRNA differentiation antagonizing non-protein coding RNA (DANCR, also known as the aforementioned ANCR) is decreased in human mesenchymal stem cells (MSCs). When DANCR is silenced, the proliferation and osteogenic differentiation of human bone-marrow-derived MSCs are significantly enhanced. Conversely, when DANCR is overexpressed, these processes are markedly inhibited. This negative control of osteogenic differentiation and proliferation by DANCR is achieved through inactivation of the p38 mitogen-activated protein kinase pathway, and DANCR has been proposed as a potential therapeutic target (43).

### LncRNA-H19

**Figure 2:**
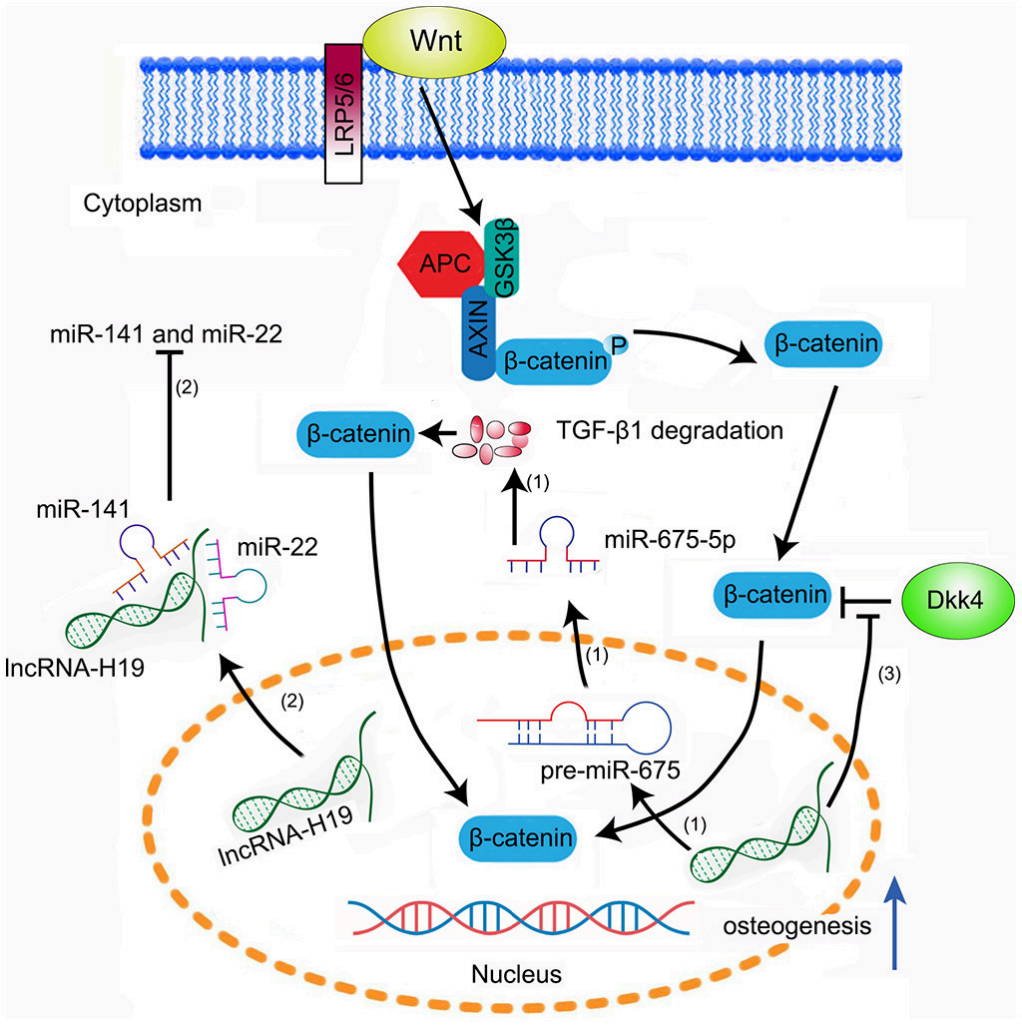
Modes of action of lncRNA-H19 in osteoblast differentiation. (1) LncRNA-H19 drives miR-675, which targets TGF-β1 for degradation. (2) LncRNA-H19 acts as an endogenous sponge for miR-141 and miR-22, which are negative regulators of osteogenesis, drawing them away from their targets and thereby causing osteoblast differentiation. (3) Binding of Wnt and its co-receptor LRP5/6 results in the inhibition of β-catenin degradation, activation of Wnt/β-catenin signaling, increased osteoblast function, and bone formation. β-catenin is incorporated into a destruction complex containing AXIN, adenomatous polyposis coli (APC), and the serine-threonine kinase glycogen synthase kinase-3 (GSK3β). LncRNA-H19 inhibits Dkk4 expression and subsequently promotes Wnt signaling, resulting in increased osteogenesis.

LncRNA-H19, a 23-kb lncRNA located on human chromosome 11, is a transcript from a conserved imprinted gene cluster that is transcribed only from the maternally inherited allele (44). Aberrant expression of lncRNA-H19 promotes its function as a pathogenetic gene in many human diseases, such as cancer, diabetes, and coronary disease. LncRNA-H19 was first identified as an osteogenesis accelerator. Huang and colleagues (45) found that the expression of lncRNA-H19 was up-regulated during osteoblast differentiation of MSCs, as evidenced by RUNX2, ALP, and OCN, and that knockdown of lncRNA-H19 significantly repressed osteoblast proliferation *in vitro*. Moreover, overexpression of lncRNA-H19 induced osteoblast differentiation of human MSCs *in vitro* and promoted heterotopic bone formation *in vivo*. Subsequently, they found that miR-675, known to be embedded in the first exon of H19, promoted osteoblast differentiation of human MSCs, due partly to the pro-osteogenic effect of lncRNA-H19–targeted TGF-β1 degradation, and that inhibition of osteogenesis required the activity of histone deacetylase (HDAC) (45). Moreover, the down-regulation of TGF-β1 inhibited phosphorylation of Smad3 and decreased the expression level of HDAC. Therefore, lncRNA-H19 is believed to regulate osteoblast differentiation via a unique regulatory mechanism involving the miR-675/TGF-β1/Smad3/HDAC pathway, although further analysis of the molecular mechanism of lncRNA-H19 in the regulation of osteoblast differentiation is required. Furthermore, lncRNA-H19 can act as ceRNA. Liang et al. (46) recently found that lncRNA-H19 regulated osteoblast differentiation by blocking miR-141 and miR-22, which are negative regulators of osteogenesis and the Wnt/β-catenin pathway, and thus that it can act as an miRNA sponge, leading to the enhancement of osteoblast proliferation. Moreover, they found that lncRNA-H19 promoted osteoblast differentiation through association with the activated Wnt/β-catenin pathway in human MSCs. They also identified a new self-regulatory feedback between lncRNA-H19 and its encoded miR-675-5p. MiR-675-5p was found to directly target lncRNA-H19 and to counteract its inhibitory effect (Figure 2).

Low expression of lncRNA-H19 has been detected in disuse osteoporosis (DOP), accompanied by the inhibition of osteogenesis and impaired trabecular bone growth (39). Bioinformatic analysis indicated that Dickkopf 4 (Dkk4) was a potential target gene of H19 and that Wnt signaling mediated the biological effects of H19 in DOP (39). Dkk4 is known to act as an inhibitor of osteoblastogenesis. Immunohistochemical analysis revealed that Dkk4 was specifically down-regulated in rats with DOP. Moreover, the three key genes in downstream Wnt signaling (c-Myc, ZEB1, and SNAIL) were dramatically down-regulated in DOP. A subsequent *in vitro* cell assay further demonstrated that lncRNA-H19 regulates osteoblastogenesis via the Dkk4/Wnt signaling cascade (39).

### LncRNA hypoxia-inducible factor 1α-anti-sense 1

Hypoxia-inducible factor 1α-anti-sense 1 (HIF1α-AS1) is an lncRNA that has been shown to play key roles in the proliferation and apoptosis of vascular smooth muscle cells *in vitro*. Xu et al. (38) found that lncRNA–HIF1α-AS1 promoted osteoblastic differentiation of human MSCs through down-regulation of homeobox D10. Moreover, they implied a potential role for lncRNA–HIF1α-AS1 in osteoblastic differentiation targeting the histone deacetylase SIRT1, a regulator of bone mass and osteoblastogenesis.

### LncRNA TSIX

LncRNA TSIX, a gene antisense of X-inactive specific transcript, has been clearly implicated in X-chromosome inactivation (47). LncRNA TSIX is believed to play an important role in the regulation of collagen expression in dermal fibroblasts. Bu et al. (47) found an up-regulated lncRNA TSIX mRNA level, down-regulated miR-30a-5p expression, and increased osteoblast apoptosis (osteoblastic cell line MC3T3-E1) in a particle-induced osteolysis model experiment. The same result was obtained in an *in vitro* experiment. They also found that lncRNA TSIX promoted osteoblast apoptosis by down-regulating miR-30a-5p. These findings have established a critical role for lncRNA TSIX in osteoblast apoptosis *in vivo* and *in vitro*. Future studies with other animal models of osteoporosis are warranted to explore the mechanism of lncRNA TSIX in osteoblast regulation.

### LncRNA maternally expressed gene 3

Maternally expressed gene 3 (MEG3), located in the chromosome 14 DLK1-EG3 imprinting region containing multiple imprinted genes, is expressed in many normal tissues. Researchers have found that lncRNA MEG3 plays a critical role in the differentiation of osteoblasts in multiple myeloma, a hematological cancer that decreases bone mass by attenuating osteoblast proliferation and osteoblastogenesis. LncRNA MEG3 expression was found to increase during osteogenic differentiation of MSCs from normal patients (48). LncRNA MEG3 knockdown significantly inhibited osteoblast differentiation, as evidenced by RUNX2, osterix, and OCN, while overexpression of lncRNA MEG3 enhanced the expression of these markers in normal MSCs (48). Moreover, lncRNA MEG3 knockdown decreased transcription of BMP4, a regulator of osteoblast maturation. Overexpression of lncRNA MEG3 or administration of exogenous BMP4 remedied the osteogenic defects of MSCs derived from patients with multiple myeloma. Molecular analysis demonstrated that lncRNA MEG3 activated BMP4 transcription by dissociation of the transcription factor SOX2 from the BMP4 promoter (48).

Conflicting results were reported in a recent publication. Wang et al. (49) findings suggest that the effect of lncRNA MEG3 in bone marrow MSCs is antiosteogenic. LncRNA MEG3 regulated osteogenic differentiation of MSCs from patients with PMOP by up-regulation of miR-133a-3p expression, inhibiting the expression of SLC39A1, a direct target molecule for miR-133a-3p, which blocked osteogenic differentiation of MSCs (49). Such discrepancies might reflect the disease-dependent effects of lncRNA MEG3 on osteoblast differentiation.

### LncRNA taurine-up-regulated gene 1

Taurine-up-regulated gene 1 (TUG1) is a 6.7-kb lncRNA located at chromosome 22q12 that prompts the proper formation of photoreceptors in the developing rodent retina (50). LncRNA TUG1 is highly expressed in human aortic valves and valve interstitial cells (51). Its knockdown results in reduced osteoblast differentiation in calcific aortic valve disease *in vitro* and *in vivo*. LncRNA TUG1 regulates the expression of the RUNX2 gene by sponging of miR-204-5p, thereby promoting osteogenic differentiation in calcific aortic valve disease (51). He et al. (52) showed that lncRNA TUG1 enhances osteogenic differentiation of periodontal ligament stem cells through the activation of homolog A, an RNA-binding protein. Additional validation of the role of lncRNA TUG1 in the skeletal system is needed.

### LncRNA metastasis-associated lung adenocarcinoma transcript-1

Metastasis-associated lung adenocarcinoma transcript-1 (MALAT1) is a highly conserved and ubiquitously expressed lncRNA, located in chromosomal region 11q13 with a length of 8700 nucleotides. Recent research has demonstrated that lncRNA MALAT1 can cause osteoblast differentiation of human aortic-valve interstitial cells by sponging of miR-204 (53). Gao et al. (54) showed that lncRNA MALAT1 increases osterix expression in human MSCs, thereby causing osteogenic differentiation by competitive binding to miR-143 to inhibit miR-143 expression. However, the function of lncRNA MALAT1 in human MSC differentiation and osteoporosis remains unknown.

### LncRNA linc-ROR

Linc-ROR, a 2.6-kb lncRNA consisting of four exons and located on chromosome 18, was discovered in 2010 (55). It is a key regulator of pluripotent stem cell reprogramming, and its up-regulation has been associated with the osteogenic differentiation of human MSCs (56). Accordingly, lncRNA Linc-ROR knockdown blocks the osteogenic differentiation of human bone-marrow-derived MSCs. Moreover, lncRNA Linc-ROR has been found to activate osteogenic differentiation in part by acting as a sponge for miR-138 and miR-145 and by activating canonical Wnt/β-catenin signaling, as evidenced by the increased expression of transcriptional factors of c-Myc, CD44, Oct4, and cyclin D1 (56).

### LncRNA HoxA-AS3

At the transcript level, expression of the lncRNA HoxA-AS3, located in the HoxA genomic region, increases gradually during adipogenic differentiation of human MSCs (57). Moreover, lncRNA HOXA-AS3 interacts with EZH2 to recruit it to the promoter region of RUNX2 and causes subsequent suppression of RUNX2 expression and osteogenesis. Thus, lncRNA HOXA-AS3 might play a role in the osteo-adipogenic lineage commitment of MSCs and the adipogenic transdifferentiation of osteoblasts (58).

### Other lncRNAs involved in osteogenic differentiation

Cui et al. (59) demonstrated that the knockdown of lncRNA NONHSAT009968 could accelerate the osteogenic differentiation of MSCs in an inflammatory environment by inhibiting the expression of staphylococcal protein A. Using comprehensive lncRNA profiling, Wang et al. (60) found that human PDLSC osteogenesis impairment–related lncRNA (lncRNA-POIR) causes osteogenic differentiation from PDLSCs in inflammatory microenvironments.

### Key lncRNAs in osteoclasts

Osteoclasts are derived from hematopoietic stem cells or monocytes/macrophage progenitor cells with large multinucleated cells, which are responsible for bone resorption. At present, very little information about the regulation of these lncRNAs in osteoclast function and bone resorption is available. An lncRNA microarray expression dataset was generated from osteoclastogenesis in RAW 264.7 cells that were stimulated with receptor activator of nuclear factor κB ligand (61, 62). The study identified 4348 lncRNAs that were differentially expressed in pre-osteoclasts; 1643 lncRNAs were up-regulated and 2705 were down-regulated. In mature activated osteoclasts, 1896 lncRNAs were up-regulated and 2706 lncRNAs were down-regulated. Through gene ontology and Kyoto Encyclopedia of Genes and Genomes biological pathway analysis, Dou et al. (61) displayed the potential functions of differentially expression lncRNAs. However, data showing appropriate osteoclastogenesis induction were relatively weak. Moreover, no functional characterization was performed to test the requirements for these lncRNAs in osteoclastogenesis.

LncRNA-ANCR was also shown to increase the expression of interleukin-6 and tumor necrosis factor-α in blood mononuclear cells, promoting bone resorption in post-menopausal women with low bone mineral density (63). However, the authors of this study did not provide data on the blood mononuclear cells associated with osteoclast differentiation regulated by lncRNA-ANCR.

### LncRNAs involved in osteoporosis

Osteoporosis is a systemic skeletal disorder characterized by excessive bone loss and skeletal fragility. Several lncRNAs are differentially expressed in patients with osteoporosis and have been involved in the pathogenesis of this disease; however, additional studies with replication cohorts and larger samples are needed to support these findings. Using high-throughput RNA sequencing, Fei et al. (64) identified 51 lncRNAs that were differentially expressed in patients with PMOP compared with healthy controls. These lncRNAs, including LINC00963, LOC105376834, LOC101929866, LOC105374771, and LOC100506113, may contribute to the pathogenesis of PMOP by regulating the expression of nearby and co-expressed differentially expressed mRNAs and the pathway of osteoclast differentiation. Systematic analysis of the expression profiles of miRNAs, mRNAs, and lncRNAs in the mandibles of ovariectomized mice led to the identification of six differentially expressed lncRNAs (63, 65). Some of these lncRNAs might function as ceRNAs, binding to miRNA to regulate the signaling pathways in osteoporosis in this setting.

Seventy lncRNAs, 475 mRNAs, 260 circRNAs, and 13 miRNAs were found to be significantly expressed in the peripheral blood lymphocytes of patients with PMOP compared with control subjects (66). LncRNA LINC00311 has been shown to be highly expressed in an *in vivo* osteoporosis rat model (67). The authors found that lncRNA LINC00311 induced osteoclast proliferation and inhibited osteoclast apoptosis through the Notch pathway. However, more complete elucidation of the role of aberrant lncRNA expression in the pathogenesis of osteoporosis, and the pathological roles and molecular mechanisms involved, requires further investigation. Recently, Zeng et al. (68) identified two lncRNA polymorphisms located at 5q14.3 and 7q21.3, a region containing the gene encoding lncRNA growth arrest–specific transcript 5, which is associated with bone mineral density. The authors provided suggestions for subsequent functional studies of these polymorphisms in the pathophysiology of osteoporosis.

## Conclusion and perspectives

LncRNAs are increasingly recognized as key regulatory molecules of numerous biological functions. Since the existence of lncRNAs was first demonstrated in mammals, significant progress has been made in lncRNA research. Although the bone remolding related to the disruption of lncRNA functions provides evidence for their biological relevance, the exact mechanisms of this process are not fully understood. Future therapies may involve the manipulation of lncRNA expression levels to regulate osteogenesis and osteoclastogenesis. However, many issues, including the targeting of lncRNA for bone remodeling and efficient delivery *in vivo* and *in vitro*, need to be addressed before lncRNAs can be used in the treatment of osteoporosis.

## Author contributions

FY, Q-YW, and R-SX conceived the project and designed experiments. XL and J-XY collected and analyzed data. All authors developed analytical tools and wrote, edited, and approved the final submission of the manuscript.

### Conflict of interest statement

The authors declare that the research was conducted in the absence of any commercial or financial relationships that could be construed as a potential conflict of interest.
